# Efficacy of scalp acupuncture for migraine: A protocol for systematic review and meta-analysis

**DOI:** 10.1097/MD.0000000000030926

**Published:** 2022-12-16

**Authors:** Che-Yeon Kim, Eui-Hyoung Hwang, In Heo, Sun-Young Park, Byung-Cheul Shin, Man-Suk Hwang

**Affiliations:** a School of Korean Medicine, Pusan National University, Yangsan, Republic of Korea; b Department of Korean Medicine Rehabilitation, Pusan National University Korean Medicine Hospital, Yangsan, Republic of Korea.

**Keywords:** migraine, protocol, randomized controlled trials, scalp acupuncture, systematic review

## Abstract

Migraine is a disorder that is prevalent worldwide. However, there is still no clear and effective treatment for migraine. Recently, acupuncture as a treatment has been attracting attention and studies have shown the value of scalp acupuncture. Therefore, this protocol for a systematic review and meta-analysis was created to assess the evidence of effectiveness and safety of scalp acupuncture in the treatment of migraine.

**Methods:**

All published randomized controlled trials (RCTs) in the following databases will be searched from their inception to September 2022: PubMed, EMBASE, the Cochrane Central Register of Controlled Trials (CENTRAL), OASIS, Korean Studies Information Service System (KISS), Korean Medical Database and NDSL, CiNii (Citation Information by NII), and the China National Knowledge Infrastructure (CNKI), without language restrictions. The data collection and analysis will be conducted independently by two reviewers. The Cochrane Collaboration tool will be used to evaluate the risk of bias by evaluating the available studies. A meta-analysis will be conducted using RevMan V.5.4 software.

**Results:**

The purpose of the proposed systemic review is to systematically assess the effectiveness and safety of scalp acupuncture for the treatment of migraine.

**Conclusions:**

To sum up, this review will assess the effectiveness and safety of scalp acupuncture for the treatment of migraine. The results of this review are expected to provide new guidelines for the treatment of migraine.

**Ethics and dissemination:**

The review and meta-analysis will not require ethical approval because personal information from individuals will not be involved. The results will be published in a peer-reviewed journal.

## 1. Introduction

Headache disorders, migraines in particular, are the most common disorders worldwide.^[1]^ Migraine is a primary headache disorder that causes considerable impairment and has a one-year prevalence of approximately 15% in the overall population.^[2]^

The primary symptom of migraine is extreme pulsing or splitting pain in one area of the head; other symptoms include vomiting, or susceptibility to both light and sound. Visual disorders that occur as flashing lights, zig-zag lines, or a temporary loss of vision, called “aura”, that precede pain are predictors of a migraine attack in approximately 30% of migraine patients. Migraine patients experience relapses triggered by a variety of factors, including stress, anxiety, hormonal changes, and dietary substances.^[3]^

According to the global burden of disease 2016, migraine is the second most prevalent neurological illness worldwide and causes more disorders than all other neurological illnesses combined.^[4]^ Additionally, the disability burden of migraine, by far the principal contributor, is concentrated in those of productive age.^[5]^ The other problem is that higher levels of migraine-induced disorder, as evaluated using the migraine disability assessment questionnaire, are related to an increased waste of healthcare resource and expenses among Americans with migraine.^[6]^

According to the American headache society consensus statement, drug therapy (triptans, NSAID), neuromodulation and biobehavioral therapy are being proposed as a treatment of migraine.^[7]^ One study reported that half of all patients were not content with their prevalent remedy with respect to reduction in the frequency of pain, and approximately 80% would contemplate the use of another remedy.^[8]^ In addition, drug-abuse headaches are ranked to be in the top-20 disorders worldwide.^[9]^ Current treatments of migraine have several unwanted side effects; therefore, migraine specialists have been deliberating about alternative migraine treatments.

Thus, as the main treatment for migraine is pharmacological therapy, acupuncture as a treatment is attracting attention. Based on the analysis of a systematic review of acupuncture as a treatment for migraine, acupuncture reduces the frequency of migraine attacks and is more effective and has fewer side effects than western medicine. However, the analysis failed to reach a definite conclusion, citing the limitation that stronger evidence is needed.^[10,11]^ Scalp acupuncture, called head acupuncture, is a combination of traditional acupuncture and neurology. It stimulates certain acupoints in the head by applying acupuncture, so as to provide a stimulus to the neurons in the brain. This is a new acupuncture treatment that is different from conventional acupuncture. Nowadays, it is widely used in clinical training. In particular, scalp acupuncture is a treatment specialized for neurological disorders, such as stroke, Parkinson’s disease, and insomnia.^[12–14]^ In addition, scalp acupuncture is notable in reducing disability in patients with migraine.^[15]^

However, the current evidence obtained from systematic reviews and meta-analyses is not enough to determine whether the efficacy and safety of scalp acupuncture as a treatment for migraine is sufficient compared to other treatments. The importance of research on migraines that reduce the quality of life and cause economic burden needs to be highlighted. Therefore, this article reports a protocol for a systematic review for evaluating the evidence on the efficacy and safety of scalp acupuncture as a treatment of migraine based on the latest studies, including randomized controlled trials (RCTs).

## 2. Methods

This systematic review protocol has been registered with the International Prospective Register of Systematic Reviews. The PROSPERO registration number is: CRD42022348879. The protocol will refer to the Preferred Reporting Items for Systematic reviews and Meta-Analysis Protocols.^[16]^

### 2.1. Research questions

The research question is if there is evidence regarding the efficiency and safety of scalp acupuncture for the treatment of migraines.

### 2.2. Eligibility criteria

#### 2.2.1. Types of studies

We will include studies with RCTs using scalp acupuncture to treat migraines.

#### 2.2.2. Participants

We will include participants who, regardless of their age, race, or sex, have been diagnosed with migraine (no limitation of criteria), including migraines with or without aura, acute migraines, chronic migraines, and episodic migraines and those who have received treatments, including scalp acupuncture in the study.

#### 2.2.3. Intervention

Scalp acupuncture will be used as the main intervention.

#### 2.2.4. Comparison

The comparison group will consist of patients undergoing other treatments, such as medicine and physical therapies, placebo/sham treatments, or no treatment.

#### 2.2.5. Outcome measures

##### 2.2.5.1. Primary outcome

The primary outcome will be the intensity of headache and frequency of migraines.

##### 2.2.5.2. Secondary outcome

Other outcomes will include the efficacy rate and the safety of scalp acupuncture.

### 2.3. Search strategy

All published RCTs in the following databases will be searched from their inception to September 2022: PubMed, EMBASE, the Cochrane Central Register of Controlled Trials (CENTRAL), OASIS, Korean Studies Information Service System (KISS), Korean Medical Database and NDSL, CiNii (Citation Information by NII), and the China National Knowledge Infrastructure (CNKI), without language restrictions. The search strategy that will be applied in the PubMed database is shown in Appendix A (Supplemental Digital Content, http://links.lww.com/MD/H893).

### 2.4. Data collection and analysis

#### 2.4.1. Data extraction and management

**Figure 1.**
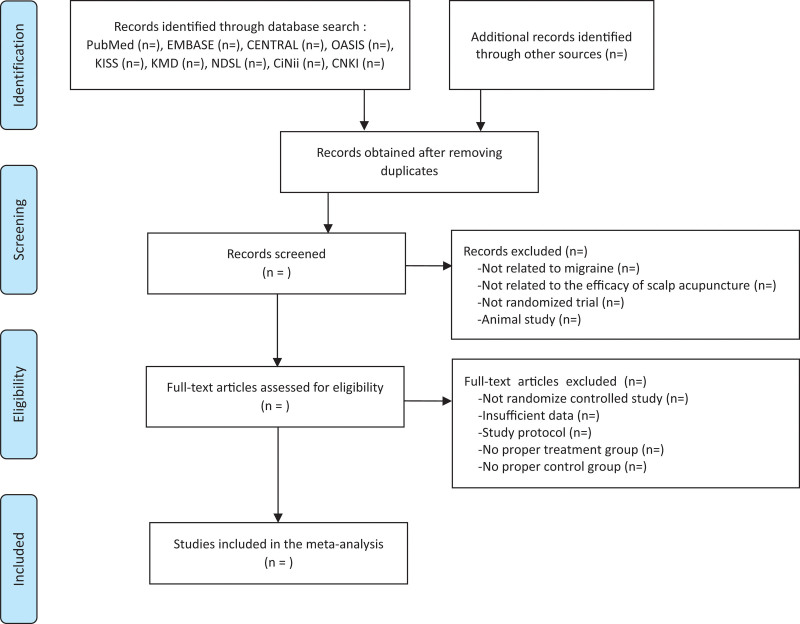
Flowchart of study selection process.

After excluding studies based on title and abstract review, two independent reviewers (CYK and MSH) will read the complete texts of each study to elicit, analyze, and organize data on the study type, participants, applied interventions, control group interventions, outcomes, and other factors. If necessary, we will contact authors for additional data. The study screening system is shown using the PRISMA flow chart in Figure 1.

#### 2.4.2. Risk of bias assessment

The assessment of the risk of bias will be executed by two independent reviewers (KCY and HMS), using the Cochrane Collaboration’s “Risk of bias” tool. The study bias will be classified as either “unclear,” “low,” or “high” risk for the following criteria: random sequence generation, allocation concealment, blinding of participants, blinding of outcome evaluation, selective outcome reporting, and deficient outcome data. Although there are seven domains, we plan to evaluate only six domains, excluding “other bias.”

#### 2.4.3. Measures of treatment effect

Odds ratio and relative risk are the primary tools used for dichotomous data of outcomes to assess the treatment effect. For continuous outcomes, the standard mean differences or weighted mean differences will be analyzed. All data, including dichotomous and continuous data, will be represented with mean ± 95% confidence intervals.

#### 2.4.4. Data synthesis

The RevMan V.5.4 (Cochrane) software will be used as the primary tool for meta-analysis. An established effect model will be adopted in the case of no statistical heterogeneity to perform a quantitative synthesis. On the other hand, in the case of meaningful statistical heterogeneity, an additional analysis will be conducted to identify the reasons for the heterogeneity. When there is obvious heterogeneity, the study will be excluded, and subsequently, a meta-analysis will be conducted using a random-effect model. Statistical heterogeneity will be evaluated by using the Cochrane’s *Q* test. A *P* value ≤ .1 will be considered as heterogeneous.

#### 2.4.5. Subgroup analysis and investigation of heterogeneity

During clinical research, subgroup analyses will be conducted in the cases of meaningful heterogeneity. The subgroup criteria may involve the control group intervention, severity of migraine, and the different types of scalp acupuncture (electrical stimulus at scalp points, bloodletting at scalp points, and embedding therapy on head points). If a meaningful difference between subgroups is identified, the results will be provided for subgroups individually.

#### 2.4.6. Sensitivity analysis

A sensitivity analysis will be performed to evaluate the compactness of the results. In studies that have a low or high risk of bias, some concerns of bias will be removed from all the included reviews.

#### 2.4.7. Grading the quality of evidence

The certainty of evidence for all data will be evaluated using the grading of recommendations assessment, development, and evaluation approach. Several factors, such as study limitations, indirectness of results, and reporting bias will be considered to evaluate the certainty of evidence. The certainty of evidence will be estimated as high, moderate, low, and very low.

## 3. Discussion

Studies have proven that scalp acupuncture can be effectively applied to the treatment and prophylaxis of migraine by reducing the pain and relapse rate.^[15,17]^ However, these studies have not been systematically organized with regard to the exact mechanism of the efficacy of scalp acupuncture. Thus, in order to assess the efficacy and safety of scalp acupuncture as a treatment of migraine, it is necessary to organize evidence, especially evidence that has been accurately proven by RCTs, so that it can be used.

The purpose of the proposed review is to systematically assess the efficacy and safety of scalp acupuncture in reducing the disability of patients with migraine. The results of this study are anticipated to present new guidelines for the treatment of migraine to clinicians and patients and thus reduce the lack of clarity regarding the treatment that remains despite the variety of drug treatments. In conclusion, this study will be the first to prove and assess the effect of scalp acupuncture for the treatment of migraines.

## Author contributions

MSH and BCS designed the study. EHH, IH, and SYP developed the search strategy. MSH and CYK conducted meta-analysis, evaluated the risk of bias and grading the quality of evidence, and wrote the manuscript. All authors provided critical revisions of the protocol and approved the final manuscript.

**Methodology:** Byung-Cheul Shin, Man-Suk Hwang.

**Project administration:** Eui-Hyoung Hwang, In Heo, Sun-Young Park.

**Supervision:** Byung-Cheul Shin, Man-Suk Hwang.

**Writing – original draft:** Che-Yeon Kim.

**Writing – review & editing:** Man-Suk Hwang.
